# Functional implications of aging-related lncRNAs for predicting prognosis and immune status in glioma patients

**DOI:** 10.18632/aging.203944

**Published:** 2022-03-10

**Authors:** Guangying Zhang, Yanyan Li, Na Li, Liang-Fang Shen, Zhanzhan Li

**Affiliations:** 1 Department of Oncology, Xiangya Hospital, Central South University, Changsha, Hunan Province 410008, PR China; 2 Department of Nursing, Xiangya Hospital, Central South University, Changsha, Hunan Province 410008, PR China; 3 National Clinical Research Center for Geriatric Disorders, Xiangya Hospital, Central South University, Changsha, Hunan Province 410008, PR China

**Keywords:** aging, lncRNA, glioma, prognosis, immune filtration

## Abstract

This study is aimed to establish a new glioma prognosis model by integrating the aging-related lncRNA expression profiles and clinical parameters of glioma patients enrolled in the Chinese Glioma Genome Atlas and The Cancer Genome Atlas. The aging-related lncRNAs were explored using Pearson correlation analysis (|R|> 0.6, *P* < 0.001), and the prognostic signature in glioma patients was screened using univariate cox regression and least absolute shrinkage/selection operator regression. Based on the fifteen lncRNAs screened out, we divided the glioma patients into three subtypes, and developed a prognostic model. Kaplan-Meier survival curve analysis showed that low-risk patients survived longer time than high-risk patients. Principal component analysis indicated that the signature of aging-related lncRNAs was clearly distinct between the high- and low-risk groups. We also found the fifteen lncRNAs were closely correlated with 119 genes by establishing a co-expression network. Kyoto Encyclopedia of Genes and Genomes analysis displayed that the high- and low-risk groups were enriched in different functions and pathways. Different missense mutations were observed in the two groups, and the most frequent variant types were single nucleotide polymorphism. This study demonstrates that the novel aging-related lncRNAs signature has an important prognosis prediction ability and may contribute to individualized treatment for glioma.

## INTRODUCTION

Glioma, especially glioblastoma, is a largely heterogeneous tumor with high recurrence and mortality rates in the central nervous system. The median survival time of World Health Organization (WHO) grade III glioma is about 3 years, whereas WHO grade IV glioma has a grave prognosis of less than 15 months [1]. The conventional treatments for gliomas are surgical resection, radiotherapy and temozolomide chemotherapy. However, the drug treatment of glioma remains challenging, because the unique structure of the blood-brain barrier prevents numerous antitumor drugs from entering the brain [2, 3]. Though various cancer therapies have been applied over the past decades, the prognosis of glioma patients remains dismal and is inconsistent even at the same tumor grade. Recently, many mutations have been proved capable of assessing the risk and predicting the prognosis of glioma, such as isocitrate dehydrogenase (IDH) mutation and 1p19q co-deletion, which present relatively favorable survival [4]. However, some of these indicators are not very comprehensive in predicting the prognosis of all-grade glioma patients, such as IDH (since nearly 80% of patients with low-grade glioma have IDH mutations). Therefore, a robust prognostic model is urgently needed to predict the survival of patients with the malignant growth and high relapse rate of glioma.

Long non-coding RNAs (lncRNAs), which are more than 200 nucleotides in length, do not encode proteins and are involved in post-transcriptional modulation and gene translation [5]. The diversity of lncRNAs is implicated in many biological functions, such as epigenetic regulation, tumor microenvironment and cell apoptosis [6]. Reportedly, overexpression of lncRNA AGAP2-AS1 can enhance breast tumor growth and trastuzumab resistance [7]. High expression of HOTAIR also promotes breast tumor cell proliferation and tamoxifen resistance [8]. Additionally, the depletion of lncRNA AGAP2-AS1 depresses proliferation and invasion, and induces apoptosis in U251 cells [9]. Moreover, ATB overexpression predicts a poor prognosis in colorectal cancer [10]. As relevant research continues, accumulating research has identified lncRNAs to play critical roles in regulating cell proliferation, invasion, apoptosis, and drug resistance in various tumors [11, 12]. Meanwhile, the vital roles of lncRNAs in degenerative diseases of the central nervous system are becoming evident [13]. LncRNAs also promote tumor immune evasion; for example, NKILA escapes immunological destruction by sensitizing T cells and inhibiting NF-κB activity [14].

As is well-known, neurodegenerative disorders and cancers being age-related diseases. Recent research demonstrates that lncRNA expression profiles influence aging. Studies suggest that aging is a significant risk factor for cancer development [15, 16]. Aging is a set of functional and structural alterations in the immune system and can reduce human immunity. Aging is manifested as a decreased ability to fight infection, a diminished response to vaccination, an increased incidence of cancers, and higher prevalence of autoimmunity and constitutive low-grade inflammation [17]. In addition to cell-intrinsic changes in both innate and adaptive immune cells, alterations in the stromal microenvironment in primary and secondary lymphoid organs are also critical in age-associated immune dysfunction [18]. Immunosenescence is a structural and functional decline in the immune system that involves organs, cells, immune factors, and regulatory networks. Decreased immunity and immune clearance, caused by immunosenescence, are key contributors to tumorigenesis [19]. However, the role of aging-related lncRNAs in gliomas has not been fully elucidated. In the present study, we integrated the gene matrix and clinical parameters from Chinese Glioma Genome Atlas (CGGA) and The Cancer Genome Atlas (TCGA). Fifteen aging-related lncRNAs were screened out from Cox regression analyses. An aging-related lncRNA prognosis model was built and used as a potential prognostic indicator to predict the prognosis of glioma patients. The differences of immune filtration were also found between risk groups established by aging-lncRNAs. Our results may provide crucial implications in clinical targeted therapy.

## MATERIALS AND METHODS

### Data sources

The complete RNA-sequencing data and corresponding clinical features of glioma patients were obtained from CGGA and TCGA (https://cancergenome.nih.gov/). The lncRNA and protein-coding genes were classified according to the gene annotation in the GENCODE project (https://www.gencodegenes.org/) from our downloaded raw readings and fragments per kilo-base of transcript per million data. Clinicopathological details (e.g., age, gender, WHO grade, radiotherapy and chemotherapy status) and survival data were obtained for further analysis. Similarly, the corresponding 1p19q codeletion and IDH mutation status were downloaded from CGGA. Glioma patients who died for non-cancer related reasons and with survival time less than 30 days were excluded. In addition, a portion of the glioma subjects with incomplete information were eliminated. No specific ethical approval or patient informed consent was required because all these data were publicly available.

### Identification of aging-related lncRNAs

The list of aging-related genes was acquired from Human Ageing Genomic Resources (HAGR; Supplementary Table 1). Correlations between the aging-related genes and lncRNAs were determined using Pearson correlation analysis on R 3.6.3. The aging-related lncRNAs were selected on basis of the criteria of correlation coefficient |R|> 0.6 and *P* < 0.001. The candidate aging-related lncRNAs identified according to the above criteria were further analyzed.

### Clustering analysis of aging-related lncRNAs

The expression patterns of these aging-related lncRNAs in different glioma patients were visualized in CGGA using principal component analysis (PCA) and validated in TCGA. The samples were clustered using the ConsensusClusterPlus algorithm and then the patients were divided into subtypes. The aging-related lncRNAs were identified from three subtypes in CGGA.

### Development and validation of aging-related lncRNAs prognostic signature

To identify the potential prognostic lncRNAs, we analyzed the association between aging-related lncRNAs and overall survival (OS) using univariate Cox regression analysis. Then the least absolute shrinkage and selection operator (LASSO) Cox regression was performed to filtrate more meaningful prognosis-related lncRNAs to establish the risk signature. Finally, we developed a prognostic signature involving fifteen aging-related lncRNAs for glioma patients. According to the risk coefficient and the expression level of each lncRNA, the risk score of each patient was calculated as: risk score = lncRNA1β × Expression + lncRNA2β × Expression + … lncRNAnβ × Expression.

### Prediction analysis of prognostic signature

With the median risk score as the threshold, the glioma patients were divided into high- and low-risk cohorts. We depicted a survival curve between the two cohorts using the Kaplan-Meier method with a two-sided log-rank test. Univariate Cox regressions were utilized to evaluate the effects of clinicopathological variables on the survival of glioma patients, including age, gender, tumor grade, radiotherapy and chemotherapy status. Furthermore, multivariate Cox regressions was performed to determine whether the risk score was an independent prognostic factor. In addition, stratified survival analysis was conducted to detect the prognostic values of the risk score model in different glioma subgroups. To further delve into the effect of single aging-related lncRNA on glioma patients in the prognostic risk model, we assessed the relationship between the expression of each lncRAN and clinical characteristics through Student’s *t*-test or one-way analysis of variance. The predictive efficiency of the risk score was generated by calculating the area under the curve (AUC) of receiver’s operating characteristic (ROC).

### Co-expression network and gene set enrichment analysis (GSEA)

Pearson correlation coefficients between the lncRNAs and aging genes in glioma patients were calculated on R 3.6.3 (R > 0.6, *p* < 0.001). The aging-related lncRNAs and a target gene co-expression network were constructed on Cytoscape 3.8.2. The co-expression of mRNAs and lncRNAs was visualized on a Sandkey diagram to show the risk type. To explore the functional enrichment of the fifteen lncRNAs, we conducted GSEA; (v.4.0.3) to determine the biological functions and pathways by the priori defined lncRNAs and verified the significant differences between the high-risk and low-risk groups.

### Immune infiltration analysis

We determined the immune cell infiltration score of each glioma patient and thereby compared the degrees of immune cell infiltration between the low- and high-risk groups. Then the difference in a proportion of 22 immune cell subtypes between the low- and high-risk groups was assessed. ESTIMATE was applied to compare the estimation, immune and stromal scores between the two groups [a]. Furthermore, the scatter plot showed the correlations of the risk score with Macrophages M0, Monocytes, and NK cells activated. The correlation between immune cell infiltration and risk scores was calculated by Pearson correlation at the significant level of *P* < 0.05.

### Somatic mutation analysis based on risk score

We explored the genetic alterations of the high- and low- risk groups and represented as waterfall plots. The top 10 somatic mutations were screened in the two groups separately. According to different classifications, the mutations were further sorted in detail. The exclusiveness and co-occurrence of mutated genes in both groups were visualized.

### Quantitative real-time polymerase chain reaction (qRT-PCR)

We collected 7 glioma and non-neoplastic brain tissues (NBTs) from the patients who underwent surgical operation. Fresh tissue samples were frozen and stored at −80°C. This research was approved by the Ethics Committee of Xiangya Hospital, Central South University. Informed consent was acquired from all the enrolled patients. Total RNA was extracted from the tissues following the manufacturer’s instructions. A cDNA synthesis kit (TaKaRa) was utilized in reverse transcription. Then, qRT-PCR was performed following the reaction steps. The related expressions of lncRNAs were normalized to GAPDH mRNA and calculated by the 2^−ΔΔCt^ method. Sequences of forward and reverse primers were: 5′-GAGGACTCAGAGGTGGAATT-3′, 5′-CAG CCAGCTTGTAGGG-3′ (LINC00665); 5′-AC CATGCTAGAAAGCCTCCC-3′, 5′-CGTCCAGCAA GGTCCTAGAG-3′ (LINC00339); 5′-ACATCGGCAT GATGG CAGAA-3′, 5′-TCACAAAAGGCGGGACC AC-3′ (SNHG16); 5′-GCTTCCAGGG GAGAT-3′, 5′-ATCAGACTGCCTGAAGA-3′ (PAXIP1.AS2); 5′-CC TATGATT TGGCCTCTGGA-3′, 5′-GAGAGCAGCG TTCAGGAAAC-3′ (LINC00092).

### Statistical analyses

All statistical analyses were conducted using R software 4.0.5. Kaplan-Meier curves and log-rank tests were applied to evaluate the survival data among subgroups. Univariate and multivariate Cox regression analyses were used to assess the independent prognostic factors for the glioma patients. The risk coefficient of the prognostic signature was calculated by LASSO regression. Prognostic accuracy of the nomogram and the predicted signature for 1-, 3-, and 5-year OS rates was estimated from the ROC curves. Single nucleotide variation was analyzed with maftools R package. Differences between variables were evaluated by independent *t*-test. Chi-square test was performed to predict the association of the variables of clinical parameters between the high- and low risk groups. Pearson and Spearman correlation analyses were also conducted. Probability value less than 0.05 or 0.001 was regarded statistically significant.

### Data availability

Original data can be obtained from Supplementary Materials.

## RESULTS

### Identification of aging-related lncRNAs

**Figure 1:**
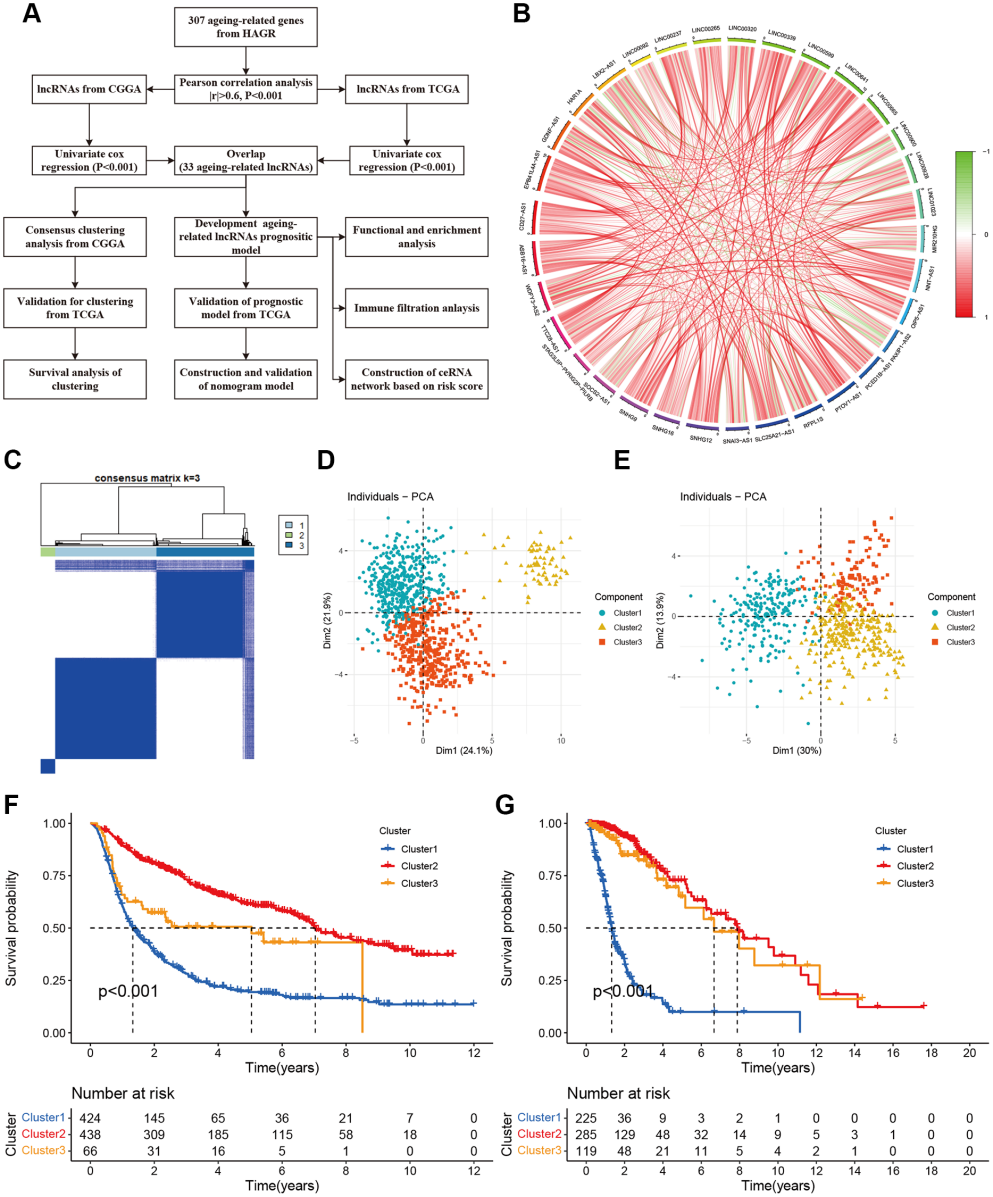
**Molecular classification based on aging-related lncRNAs.** (**A**) The flow chart of data analyses in the study. (**B**) The circle plot showed the correlation among 33 aging-related lncRNAs. (**C**) Glioma patients were divided into three clusters in CGGA. (**D**) PCA indicated that three subclasses were obtained in CGGA. (**E**) Three subclasses were validated in TCGA. (**F**) Kaplan-Meier cures of overall survival for three clusters in CGGA. (**G**) Kaplan-Meier cures of overall survival for three clusters in TCGA.

We obtained 307 aging-related genes from HAGR (Supplementary Table 1). The RNA-sequencing profiles and corresponding clinical parameters of the glioma patients were downloaded from CGGA and TCGA. Totally 928 and 629 lncRNAs were screened out from CGGA and TCGA respectively (Supplementary Tables 2 and 3). Then based on Pearson correlation analysis (|R| > 0.6 and *P* < 0.001), 226 and 152 aging-related lncRNAs were selected from CGGA and TCGA, respectively. Univariate Cox regression analysis was further performed to filtrate the potential prognostic lncRNAs from the aging-related lncRNAs, which showed 33 lncRNAs were significantly associated with the OS of the glioma patients. Details were provided in Figure 1A. The correlations among the 33 aging-related lncRNAs were presented by a circle plot (Figure 1B).

### Clustering analysis of aging-related lncRNAs associated with prognosis

To identify the aging-related glioma patterns, we classified the glioma patients into three subgroups using the 33 lncRNAs with ConsensusClusterPlus (Figure 1C). The heat map showed the sample clustering results with the optimal clustering stability (k = 3). PCA demonstrated that the glioma samples can be completely distinguished. Three subtypes were obtained in CGGA (Figure 1D) and validated in TCGA (Figure 1E). We further compared the prognosis of the three clusters according to the Kaplan-Meier cures of OS. In addition, the OS of cluster1 was shorter than the other two clusters and the prognosis of cluster2 was the best (Figure 1F and 1G). The three clusters were observably separated in CGGA (*P* < 0.001). However, there is no different distribution between cluster2 and cluster3 about survival time in TCGA.

### Establishment and validation of aging-lncRNAs prognostic model

**Figure 2:**
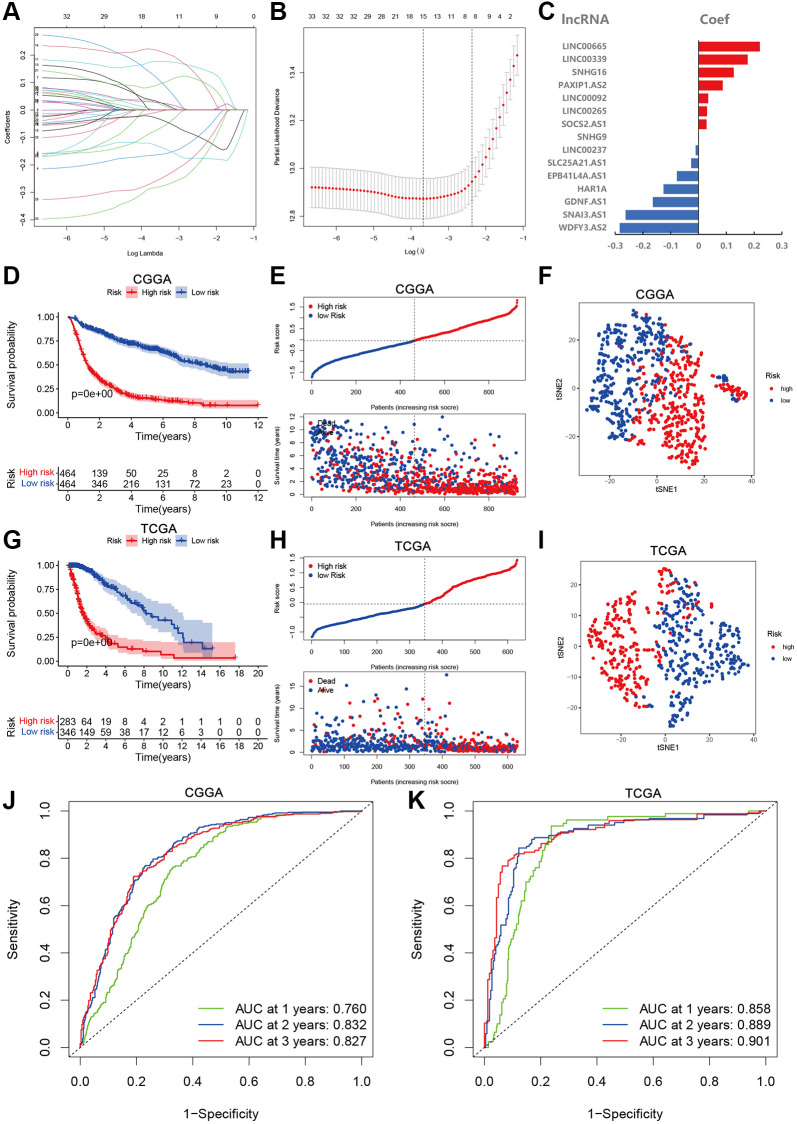
**Development and validation of aging-related lncRNAs prognosis signature.** (**A**) LASSO regression of 15 aging-related lncRNAs. (**B**) Cross-validation for tuning the parameter selection in the LASSO regression. (**C**) Coefficient of prognosis model regression. (**D**) Kaplan-Meier curves of high-risk group and low-risk group in CGGA. (**E**) Distribution of risk score and patients based on the risk score in CGGA. (**F**) The tSNE2 method showed obvious two components in CGGA. (**G**) Kaplan-Meier curves of high-risk group and low-risk group in TCGA. (**H**) Distribution of risk score and patients based on the risk score in TCGA. (**I**) The tSNE2 method showed obvious two components in TCGA. (**J** and **K**) ROC curves of prognostic signature based on risk score in CGGA and TCGA.

Based on the survival information of the glioma samples, we applied univariate Cox regression and screened 33 aging-related lncRNAs that were highly related to OS. To better explore the prognostic role of those aging-related lncRNAs in the glioma patients, we validated with LASSO Cox regression that 15 lncRNAs were most correlated with prognostics (Figure 2A). The optimal value of the penalty parameter was determined by tuning the parameter selection in the LASSO regression (Figure 2B). A risk score was calculated according to the coefficient of each lncRNA (Figure 2C). The risk scores of glioma samples were calculated as follows: risk score = (0.220 × Exp_LINC00665_) + (0.177 × Exp_LINC00339_) + (0.126 × Exp_SNHG16_) + (−0.026 × Exp_PAXIP1.AS2_) + (0.035 × Exp_LINC00092_) + (0.030 × Exp_LINC00265_) + (−0.164 × Exp_SOCS2.AS1_) + (0.002 × Exp_SNHG9_) + (−0.010 × Exp_LINC00237_) + (−0.026 × Exp_SLC25A21.AS1_) + (−0.078 × Exp_EPB41L4A.AS1_) + (−0.126 × Exp_HAR1A_) + (−0.164 × Exp_GDNF.AS1_) + (−0.262 × Exp_SNA13.AS1_) + (−0.283 × Exp_WDFY3.AS2_). With the median risk score, the glioma patients from CGGA and TCGA were divided into high- and low-risk groups. A marked difference in prognosis was found between the two groups (*P* < 0.001). Patients in the high-risk group had a worse OS than those in the low-risk group (Figure 2D, 2G). Furthermore, the risk score and survival status distributions of the glioma patients were shown in Figure 2E and 2H. The survival rate of the glioma patients was correlated with risk scores, and the mortality rate increased with a higher risk score. In addition, the tSNE2 method was used to classify the samples into two obvious components in CGGA and TCGA (Figure 2F, 2I). Besides, ROC analysis indicated that risk scores can accurately predict the prognosis of the glioma patients (1-, 2-, 3-year AUCs = 0.760, 0.832, 0.827 in CGGA, 0.858, 0.889, 0.9014 in TCGA respectively; Figure 2J, 2K). These results imply that the aging-lncRNAs prognostic model has a robust and stable prognostic value for glioma patients.

### Correlations of prognostic signature lncRNAs with clinical features

**Figure 3:**
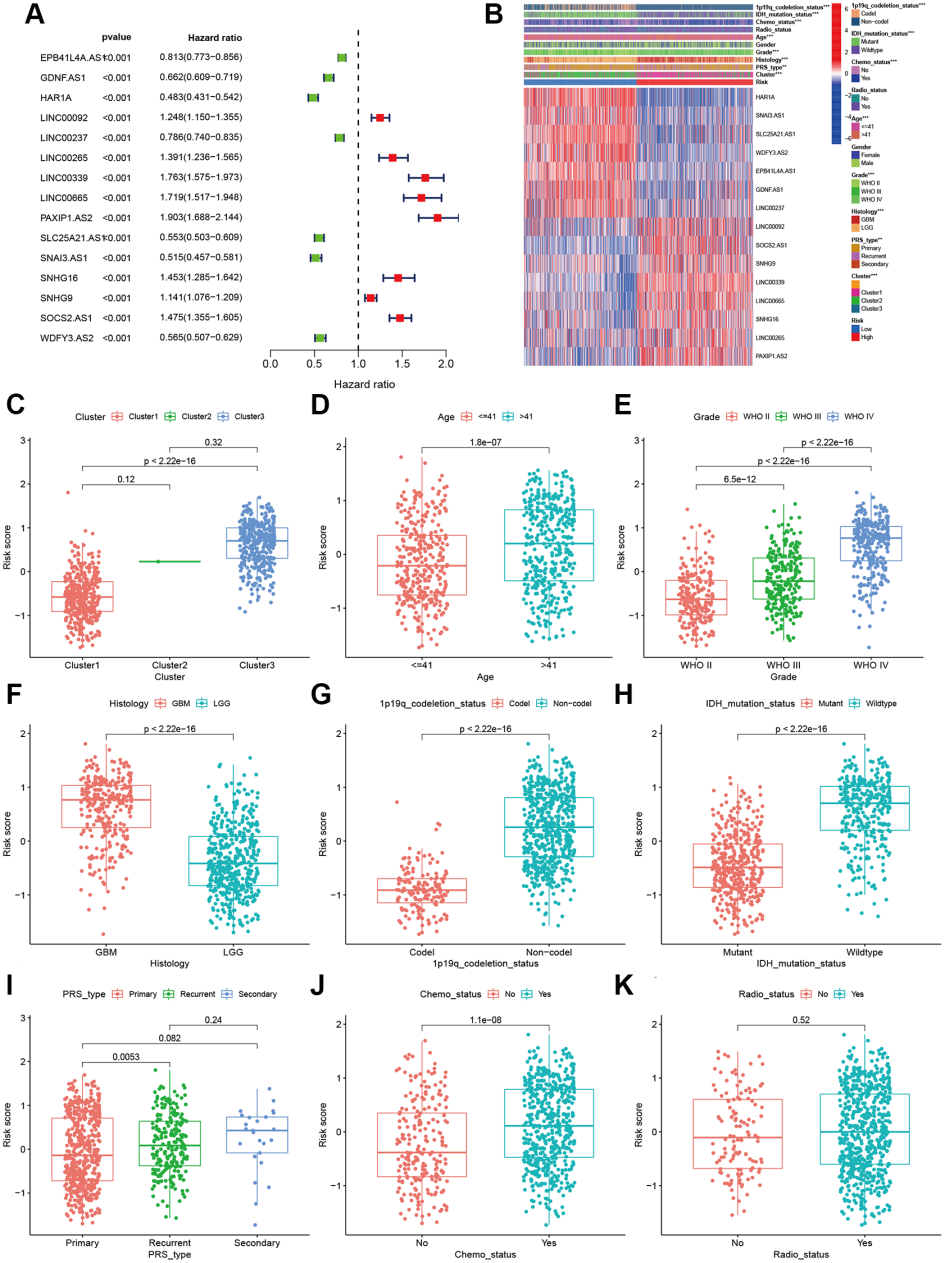
**Correlations of clinical characteristic with identified aging-related lncRNAs signature.** (**A**) Forest of univariate COX regression for 15 signature lncRNAs. (**B**) Heatmap showed that correlation of clinical parameters with risk scores and expression of 15 lncRNAs in high- and low-risk group. Boxplot showed the comparisons of risk score in different subgroups: (**C**) Cluster1 vs. Cluster2 vs. Cluster3. (**D**) age ≤41 vs. >41, (**E**) WHO II vs. WHO III vs. WHOIV. (**F**) GBM vs. LGG, (**G**) 1p19q_codeletion vs. non-codel. (**H**) IDH mutation vs. wildtype. (**I**) Primary vs. Recurrent vs. Secondary. (**J**) Chemotherapy (Yes vs. No). (**K**) Radiotherapy (Yes vs. No).

Finally, fifteen aging-related lncRNAs were involved in the prognosis signature, and their prognostic roles were evaluated using univariate Cox regression analysis. The forest plot shows that EPB41L4A.AS1, GDNF.AS1, HAR1A, LINC00237, SLC25A21.AS1, SNA13.AS1, and WDFY3.AS2 are the protective factors with HR < 1, while LINC00092, LINC00265, LINC00339, LINC00665, PAXIP1.AS2, SNHG16, SNHG9 and SOCS2.AS1 are risk factors with HR>1 in glioma patients (Figure 3A). Afterwards, we investigated the distributions of clinic pathological features and the expressions of the fifteen lncRNAs between the high- and low-risk groups, which tested whether the aging-related lncRNAs signature can predict the clinical parameters of glioma. The heat map reveals significant differences in age, grade, PRS type, histology, clusters, chemotherapy status, 1p19q codeletion and IDH mutation status between the high- and low-risk groups (*P* < 0.01; Figure 3B). Then, we further explored the relevance of risk score and each clinicopathological feature. As expected, the score of cluster 3, older age, advanced grade tumors, 1p19q non-codeletion, IDH wild type, recurrent tumors and chemotherapy status were significantly increased (Figure 3C–3K). The above results elucidate the aging-related lncRNAs signature may play a pivotal role in the tumor progression of glioma.

**Figure 4:**
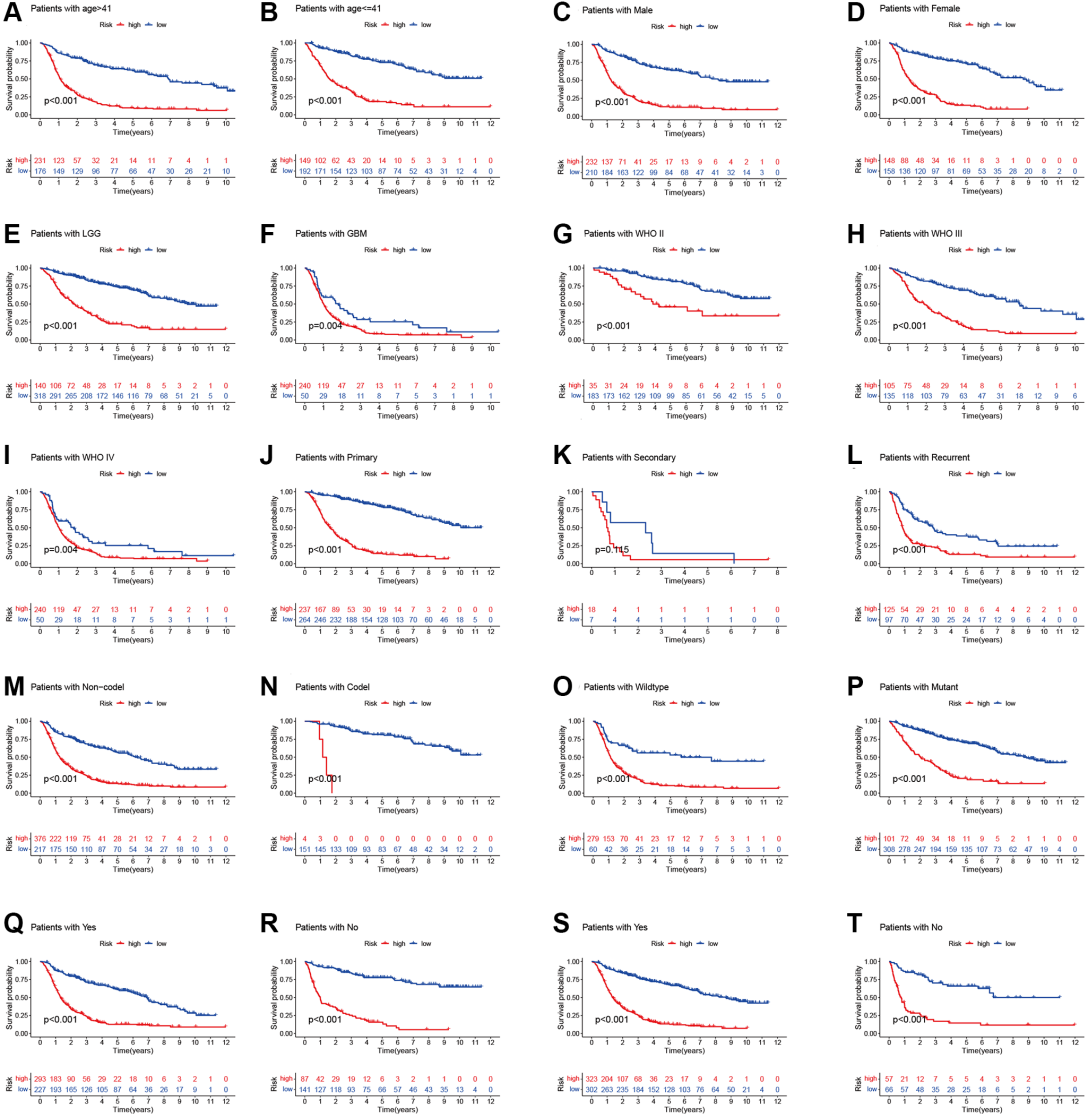
**Stratified analyses of high- and low-risk group.** (**A** and **B**) age. (**C** and **D**) Gender. (**E** and **F**) Histology. (**G**–**I**) WHO stage. (**J**–**L**) Pathology type. (**M** and **N**) 1p19q codeletion status. (**O** and **P**) IDH mutation status. (**Q** and **R**) Chemotherapy. (**S** and **T**) Radiotherapy.

To prove the applicability of our prognostic model, we performed stratification analysis to clarify whether it can evaluate prognosis in each subgroup. Kaplan-Meier survival curve analysis showed the low-risk patients had longer survival time than the high-risk patients (Figure 4). However, the OS rates were statistically similar among the patients with secondary tumor, which may be because of the smaller sample size. These results indicate that our prognosis risk signature may be a potential predictor of glioma.

### Prediction ability and independent analysis of prognostic signature

**Figure 5:**
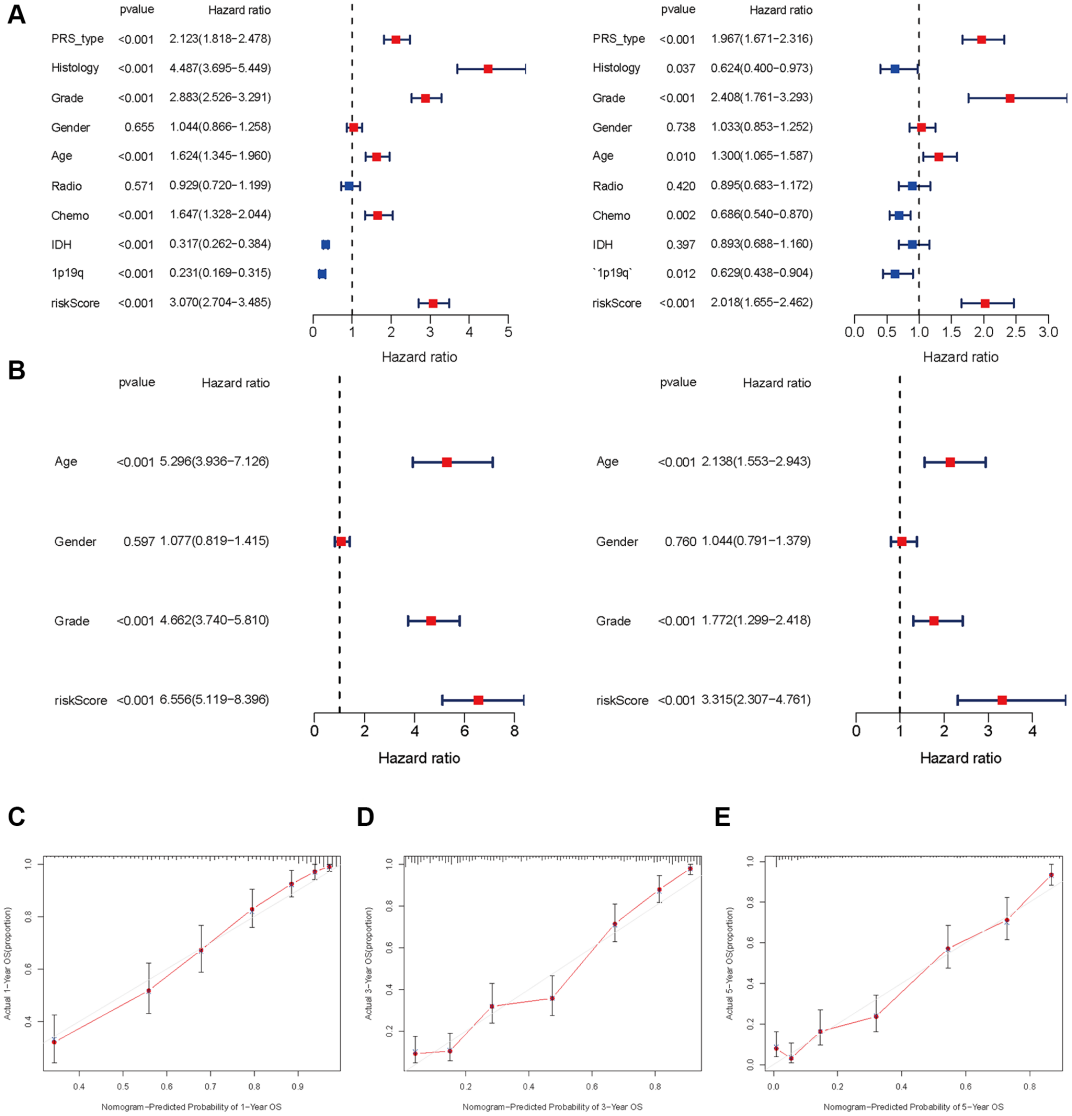
**Independent prognosis analysis of risk score.** (**A** and **B**) Univariate and multivariate cox forest plot of risk score in CGGA and TCGA. (**C**–**E**) Calibration plots of the nomogram for predicting the probability of OS at 1, 3, and 5 years in the CGGA.

**Figure 6:**
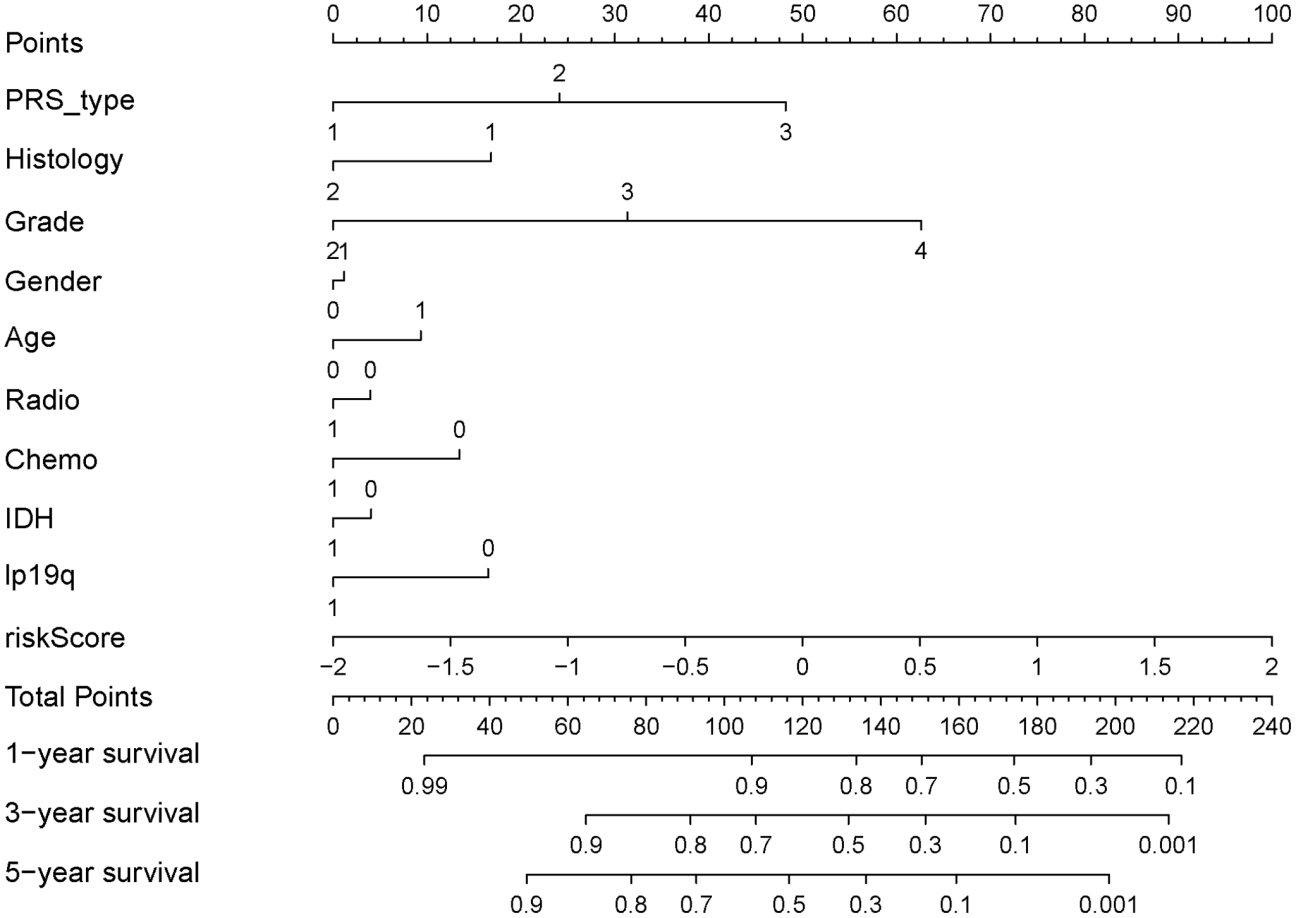
Nomograph of 1-, 3-, and 5-year overall survival probabilities predicted based on aging-related lncRNA signature.

Univariate and multivariate Cox regression analyses demonstrate that age (HR = 5.296, 95% CI: 3.936–7.1263), grade (4.662: 3.740–5.810) and risk score (6.556: 5.119–8.396) (all *p* < 0.001) are remarkably associated with OS in the CGGA training cohort. Meanwhile, similar conclusions were observed in the TCGA validating cohort (Figure 5A, 5B). To develop a clinically applicable tool by integrating the risk scores of aging-related lncRNAs prognostic signature and other clinicopathological parameters and use it to predict OS in glioma patients, we built a nomogram to evaluate the probabilities of 1-, 3- and 5-year survival. The C-index of the nomogram was 0.801 (95% CI: 0.783–0.820). Calibration curves demonstrated concordances between the actual and predicted survival rates of glioma patients after bias corrections of the nomogram in the CGGA cohort (Figure 5C–5E), and Figure 6. These results indicate that our aging-related lncRNAs prognostic signature is reliable and precise.

### lncRNA-mRNA co-expression and pathway enrichment

**Figure 7:**
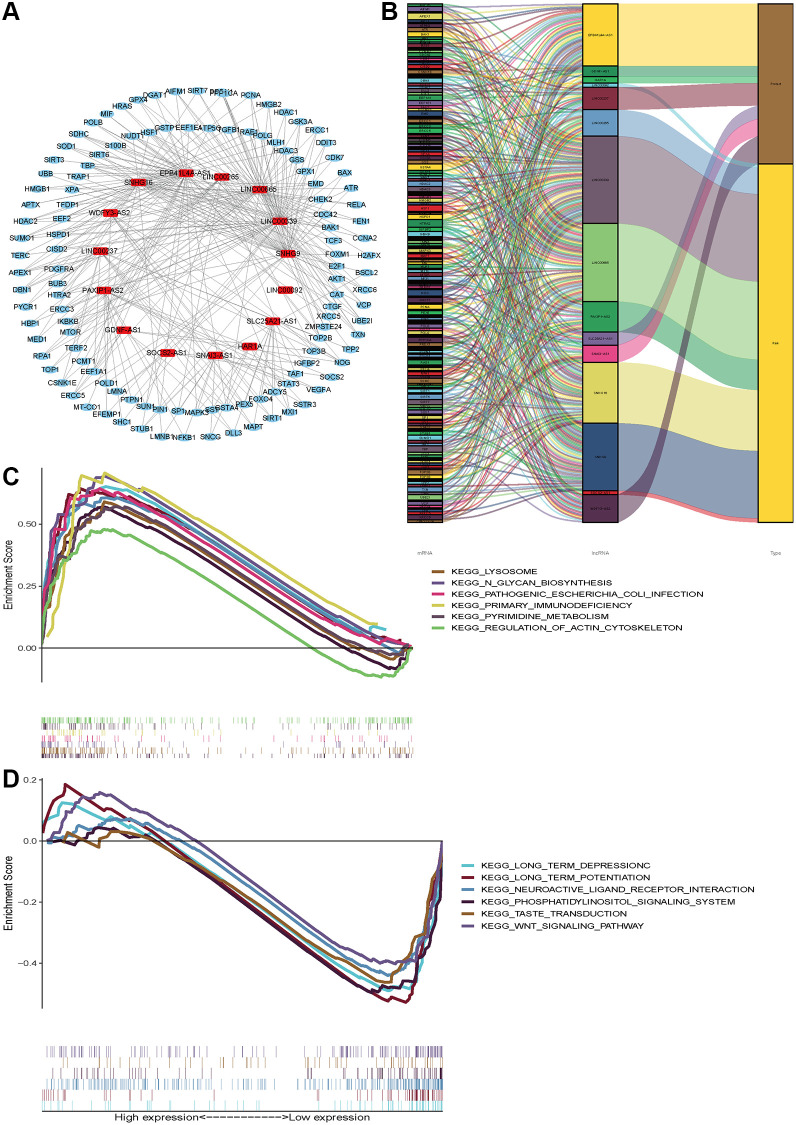
**Functional and enrichment pathways analysis.** (**A**) LncRNAs-mRNA co-expression regulatory network based-on fifteen aging-related lncRNAs. (**B**) A Sankey diagram was depicted to visualize the co-occurrences of lncRNAs, mRNAs and outcomes. (**C**) KEGG pathway enrichment analysis in high-risk group. (**D**) KEGG pathway enrichment analysis in low-risk group.

Considering that miRNA and lncRNA can affect cancer progression through mutual regulation, we explored the potential functions of the fifteen aging-related lncRNAs in glioma by establishing a co-expression network. We found the fifteen target lncRNAs were closely correlated with 119 genes, which were used to construct a complex co-expression network. The details are shown in Figure 7A. A Sankey diagram was depicted to visualize the relationship among lncRNAs, mRNAs and outcomes (risk/protective) (Figure 7B). In addition, KEGG analysis was performed to study the potential biofunction and pathway in the high- and low- risk groups. We found the high-risk group was enriched in lysosome, N glycan biosynthesis, pathogenic *Escherichia coli* infection, primary immunodeficiency, primidine metabolism and regulation of actin cytoskeleton. Moreover, the low-risk group was enriched in long-term depression, long-term potentiation, neuroactive ligand receptor, phosphatidylinositol signaling, and the WNT signaling pathway. The top six significant gene sets in the two groups were presented in Figure 7C, 7D. The above data provide valuable insights to find potential individualized treatments in different risk score groups of glioma patients in the future.

### Immune status based on risk score

**Figure 8:**
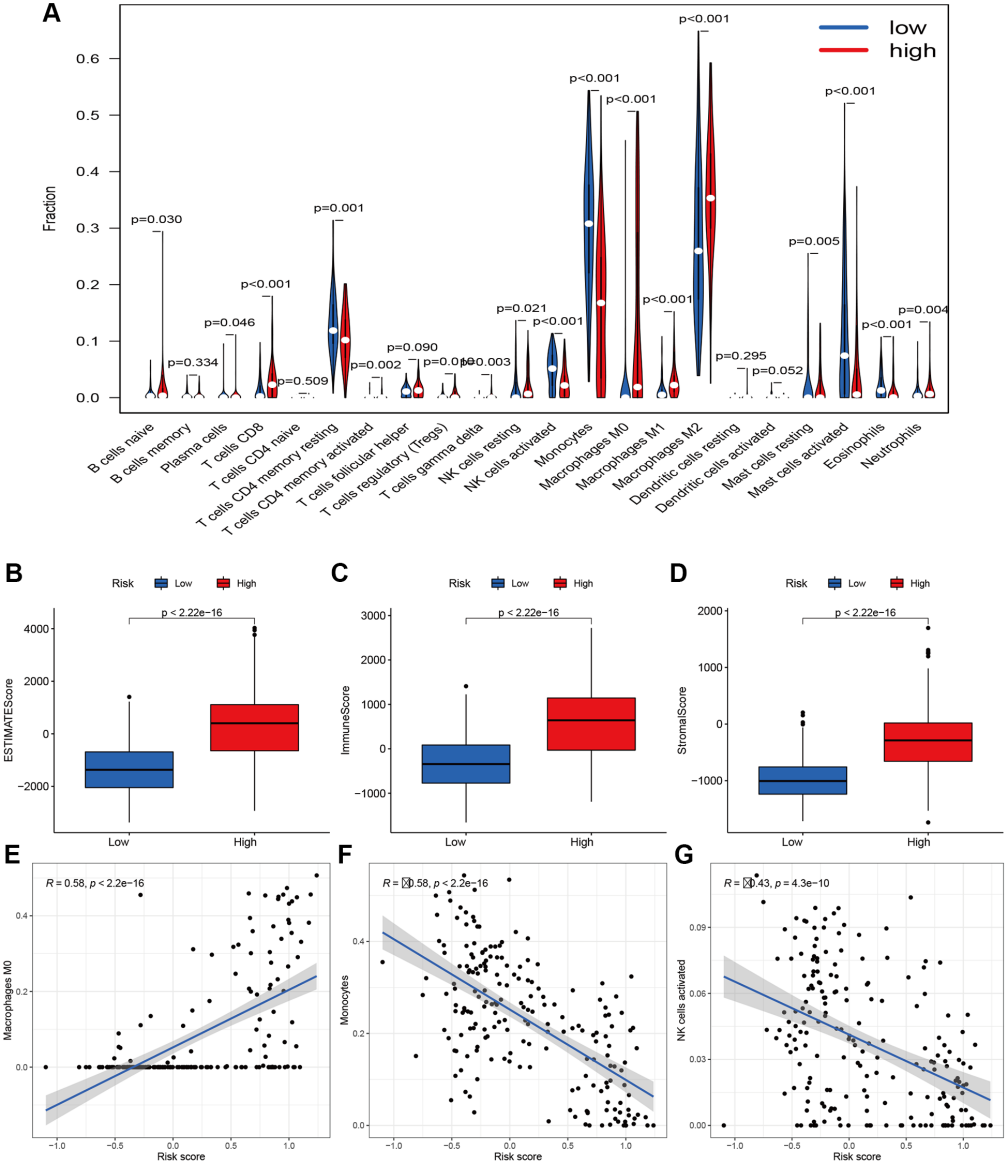
**Immune filtration analysis between high- and low-risk groups.** (**A**) Differential analysis of immune-related cells based on risk score. (**B**–**D**) Boxplot showed the comparisons of Estimation, immune and stromal score between high- and low-risk groups. (**E**–**G**) Scatter plot showed that the correlations of risk score with Macrophages M0, Monocytes, and NK cells activated

To further investigate the various infiltration of immune cells between the high- and low-risk groups, we compared the differences in infiltration of 22 immune cells between the two groups. The proportions of B cells naive (*p* = 0.030), plasma cells (*P* = 0.046), T cells CD8 (*P* *<* 0.001), T cells CD4 memory activated (*P* = 0.002), T cells regulatory (*P* = 0.001), T cells gamma delta (*P* = 0.001), NK cells resting (*P* = 0.021), Macrophages M1 and M2 (both *P* *<* 0.001), Mast cells resting (*P* = 0.005) and Neutrophils (*P* = 0.004) all increased in the high-risk group (Figure 8A). Besides, we calculated the ESTIMATE scores, immune scores, and stromal scores in the two groups by using ESTIMATE. Results indicated the scores of the high-risk group were higher than those of the low-risk group (*P* < 2.22e-16) (Figure 8B–8D). The risk scores were negatively correlated with the activation of Monocytes (R = 058, *P* = 2.2e-16) and NK cells (R = 0.43, *P* = 4.3e-10) (Figure 8F and 8G). However, the Macrophages M0 activation was upregulated with an increased risk score (Figure 8E). These findings suggest different immune infiltration statuses between the two groups.

### Somatic mutation analysis based on risk score

**Figure 9:**
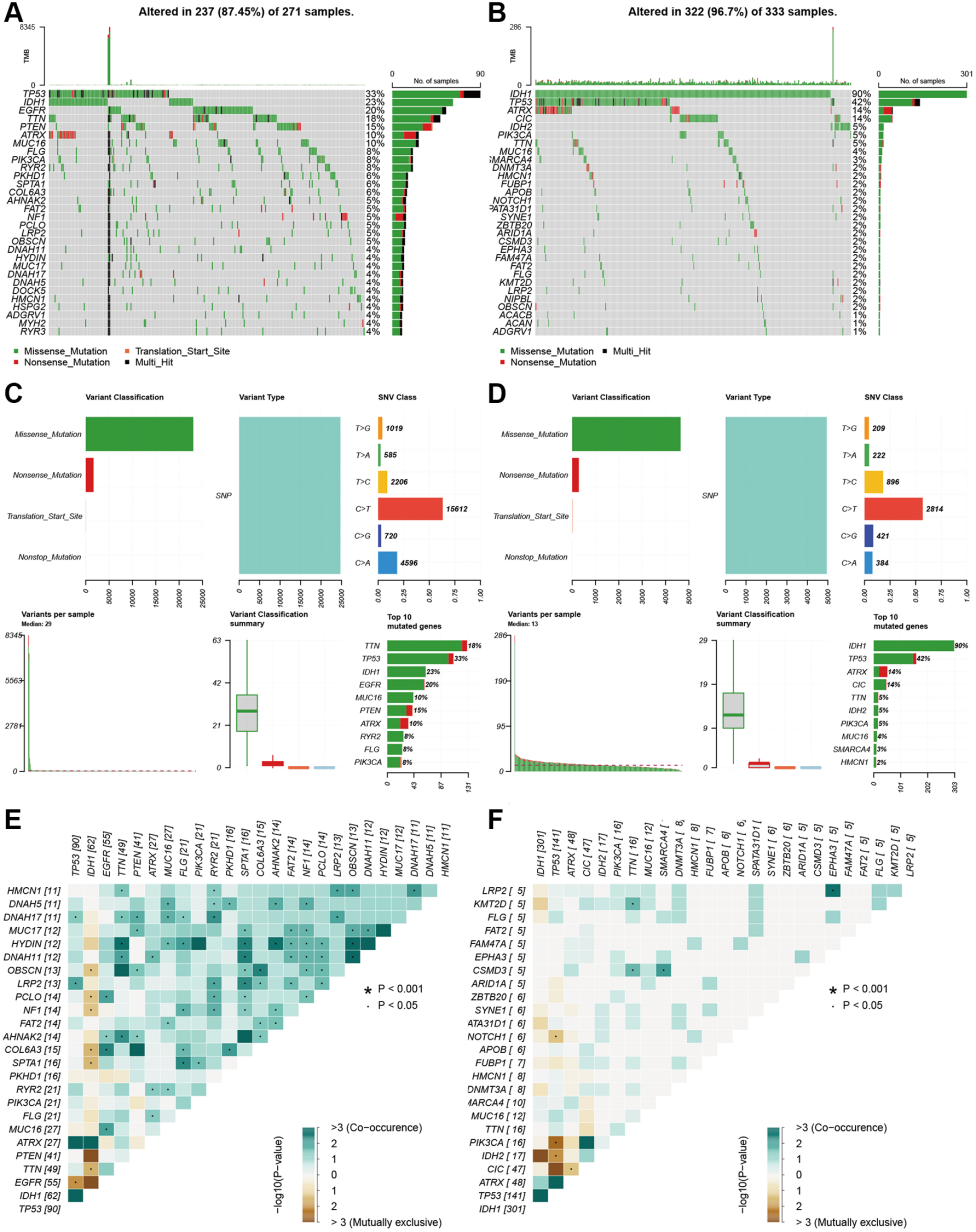
**Landscape of mutation profiles between high- and low-risk glioma patients.** (**A** and **B**) Waterfall plots showed the mutations information in each sample of high- and low-risk group glioma patients. (**C** and **D**) The variant classification in high-and the low-risk group glioma patients. (**E** and **F**) The exclusive and co-occurrence in high-and the low-risk group glioma patients.

We performed somatic mutation profiles to analyze the gene mutation in the high- and low-risk groups involving 604 glioma patients. The waterfall plots exhibit that top 10 mutated genes are TP53, IDH1, EGFR, TTN, PTEN, ATRX, MUC16, FLG, PIK3CA and RYR2 in the high-risk group, and are IDH1, TP53, ATRX, CIC, IDH2, PIK3CA, TTN, MUC16, 6MARCA4 and DNMT3A in the low-risk group (Figure 9A, 9B). Clearly, some mutated genes are at high mutation frequency in the two groups. Then the mutations were further sorted according to different classifications, The missense mutations account for the majority in both groups. The most frequent variant types are single nucleotide polymorphism (SNP), which occurred as C > T and T > C in the low-risk group and as C > T and C > A in the high-risk group (Figure 9C, 9D). Recently, co-occurrence and mutual exclusivity of genetics were often observed in cancers. The co-occurrence mutations in the high-risk group were much than those in the low-risk group (details in Figure 9E, 9F). However, they display significant exclusivity of mutations. For instance, IDH1 was mutually exclusive with PTEN and EGFR in the high-risk group, and with IDH2 in the low-risk group. This interrelated mutation suggests functional interactions, which may provide new insights into clinical treatment.

### Validation of aging-related lncRNAs expression levels

To further verify our results, we detected the expressions of five aging-related lncRNAs in 7 gliomas (4 at WHO grade II and 3 at WHO grade III) and NBTs by using qRT-PCR. Results show the mean expression levels of LINC00665, LINC00339, SNHG16, PAXIPI.AS2 and LINC00092 in the glioma tissues are higher than those in the NBTs (Supplementary Figure 1A–1E). Moreover, the higher-grade patients display higher lncRNAs expression. The above results confirm the reliability of our analysis.

## DISCUSSION

Glioma has a high recurrence rate and leads to a mortal outcome. The therapeutic effect of glioma, especially glioblastoma, remains unsatisfactory. Hence, potential prognostic indicators shall be identified for this highly heterogeneous disease. Accumulating evidences prove that lncRNAs play a pivotal role in tumor occurrence, development, metastasis and drug resistance and are novel potential biomarkers [20]. Moreover, tumor data about lncRNAs are accumulating in public databases following the wide application of high-throughput technologies and the increasing improvement of data-sharing [21]. LncRNAs modulate diverse bioprocesses and their role in aging has recently attracted much attention. LncRNAs can regulate cell senescence, telomere length, and stem cell differentiation in the aging process [22].

Aging is an inevitable process and is considered one of the predominant risk factors for most chronic diseases, including cancers [23]. Aging and cancers are interrelated. Aging-related genes can regulate cell senescence and tumor malignancy. The current view is that cell aging may promote the occurrence and development of gliomas, because gliomas are more common in the elderly, in whom the number of senescent cells increases dramatically in the brain [24]. Aging-associated genes are linked to the progression and prognosis of gliomas [25, 26]. DNA damages from radiochemotherapy can induce cell aging, which may be associated with glioma recurrence after treatment. Moreover, aging brain cells secrete excessive factors, such as MMP-2 and MMP-9, to promote cell survival and invasion [27, 28]. A seven- senescence-associated-gene signature was established to predict the overall survival of Asian patients with hepatocellular carcinoma [29]. Senescence-associated genes were recognized using two senescent cell models, and we identified aging-related genes based on previous research. The genes used in previous research did not appear in the list of aging genes from HAGR. There are remarkable differences between our study and the previous study. At present, studies on the indicators of glioma based on the lncRNA signature are mounting. However, the potential role of the aging-related lncRNA prognosis signature in glioma is inadequately studied. Hence, our study is aimed to assess the role of aging-related lncRNAs in glioma using CGGA as the training cohort and TCGA as the validation cohort.

We identified a risk signature of fifteen aging-related lncRNAs in gliomas through uni-cox regression and LASSO analysis. Finally, eight lncRNAs (LINC00092, LINC00265, LINC00339, LINC00665, PAXIP1.AS2, SNHG16, SNHG9 and SOCS2.AS1) were found associated with high risk, and patients with high expressions of these lncRNAs had unfavorable prognosis. The remaining seven lncRNAs (EPB41L4A.AS1, GDNF.AS1, HAR1A, LINC00237, SLC25A21.AS1, SNA13.AS1, and WDFY3.AS2) were related to low risk. The AUCs to predict the 1-, 2- and 3-year OS rates of glioma in the training cohort are 0.760, 0.832, and 0.827 in CGGA and are 0.858, 0.889, and 0.9014 respectively, indicating that the prognostic risk model is reliable and stable. Recently, many studies reveal the important role of lncRNA as oncogenes or cancer suppressor genes in various tumors. LncRNAs reportedly play a complex regulatory role in tumor progression. Zhao et al. found that LINC00092- silenced cells presented obviously compromised metastatic potential and lower invasive capacity in ovarian cancer, which were involved in glycolysis [30]. Moreover, LINC00665 overexpression can reverse the invasion and migration abilities through encoding micropeptide in triple-negative breast tumor cells [31]. Meanwhile, LINC00665 regulates stemness and epithelial-to-mesenchymal transition (EMT) to promote gemcitabine resistance [32]. LINC00665 in glioma cells can also inhibit tumor progression via STAU1-mediated mRNA degradation [33]. LncRNA WDFY3-AS2, as a ceRNA, inhibits invasion ability, which is correlated with lymph node metastasis and tumor - node - metastasis (TNM) stage in oesophageal squamous cell carcinoma [34]. Moreover, WDFY3-AS2 can upregulate SDC4 expression to promote cisplatin resistance in ovarian cancer, and si-WDFY3-AS2 reduces the invasion and migration of tumor cells [35].

PCA based on aging-related lncRNAs divided the glioma samples into three clusters that were significantly different in OS. Continuous inflammatory response led to cancers. In addition, the aging-related lncRNAs were related to immune cell infiltration. Our result revealed that different immune infiltration statuses emerged between the two groups of glioma patients. The risk scores were correlated negatively with the activation of Monocytes and NK cells and positively with the activation of Macrophages M0. Furthermore, we systemically analyzed the key signaling pathways of those lncRNAs by KEGG analysis. Most of the enriched pathways manifested immunomodulatory functions, and the top five significantly enriched pathways were involved in phosphatidylinositol signaling and WNT signaling. Somatic mutations are generally considered as tumor-initiating events [36]. Then, we also used waterfall plots to show the common mutation in gliomas and found TP53 and IDH1 genes were both at high frequency of mutations in the two groups. This fifteen-lncRNAs risk signature can not only effectively predict the prognosis of gliomas, but also reflects clinicopathological factors (e.g., grade, chemotherapy status, 1p19q codeletion and IDH mutation status). Hence, this signature is a potential precise indicator for the prognosis of gliomas.

In conclusion, we constructed an independent and robust prognostic signature using fifteen aging-related lncRNAs. This risk signature is based on transcriptome databases and shall be validated with some fundamental experiments. Next, further investigation is needed to clarify the underlying mechanisms and to reveal how it regulates the infiltrating immune cells in gliomas. This study may be beneficial for clinicians to identify high-risk patients more accurately and to improve the prognosis of gliomas.

## Supplementary Materials

Supplementary Figure

Supplementary Tables 1-3
