# Supplementary material for: Functional implications of aging-related lncRNAs for predicting prognosis and immune status in glioma patients
Source: Aging (Albany NY). 2022 Mar 10;14(5):2348–66. doi: 10.18632/aging.203944 (PMC8954967; doi:10.18632/aging.203944)
Supplement: Supplementary Figure [file aging-14-203944-s001.pdf]

SUPPLEMENTARY FIGURE

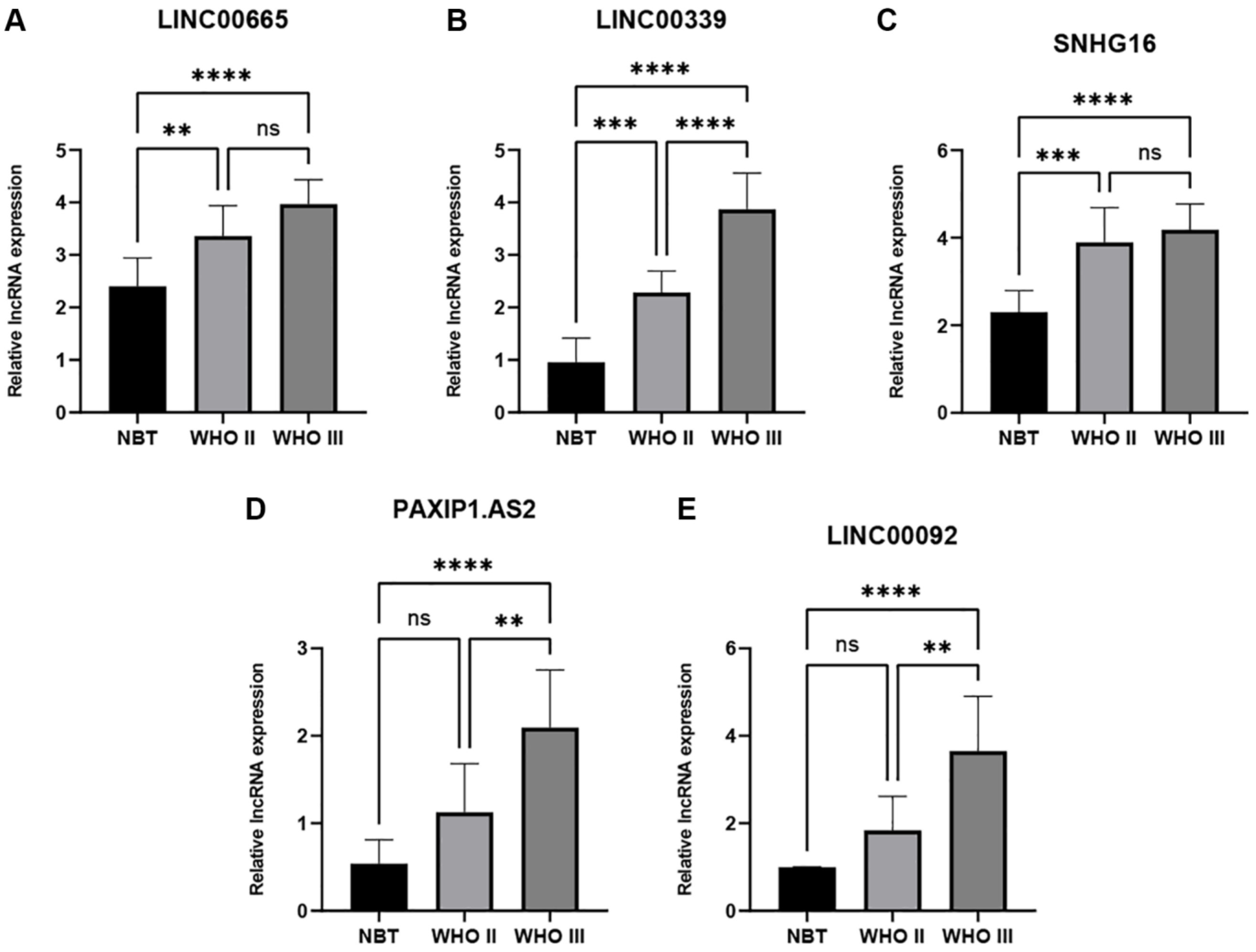

Supplementary Figure 1. Validations of several identified lncRNAs in glioma tissue. (**A**–**E**) LINC00665, LINC00339, SNHG16, PAXIPI.AS2, LINC00092.
